# Application of Microwave Ablation Combined With Chai Hu Qing Gan Tang in the Treatment of Idiopathic Granulomatous Mastitis

**DOI:** 10.1155/tbj/2731494

**Published:** 2025-07-06

**Authors:** Hang Li, Bing Li, Haiying Chen, Xiaoli Liu, Hongling Wang, Guoliang Zhang

**Affiliations:** ^1^Department of Breast Surgery, Affiliated Hospital of Putian University, Putian 351100, Fujian, China; ^2^Department of Medical Oncology, Affiliated Hospital of Putian University, Putian 351100, Fujian, China; ^3^The Third Clinical Medical College, Fujian Medical University, Fuzhou 350000, Fujian, China; ^4^Department of Pathology, Affiliated Hospital of Putian University, Putian 351100, Fujian, China; ^5^Department of General Surgery, Xiamen Xinkaiyuan Hospital, Xiamen 361000, Fujian, China; ^6^Department of Thyroid Surgery, Affiliated Hospital of Putian University, Putian 351100, Fujian, China

**Keywords:** Chai Hu Qing Gan Tang, cyber pharmacology, idiopathic granulomatous mastitis, microwave ablation, traditional Chinese medicine treatment

## Abstract

**Objective:** To investigate the efficacy of microwave ablation (MVA) combined with Chai Hu Qing Gan Tang (CHQGT) for idiopathic granulomatous mastitis (IGM).

**Methods:** 480 patients were divided into the CHQGT combination group (CHQGT + MVA), corticosteroid combination group (glucocorticoids + MVA) and control group (glucocorticoids), with 160 cases in each group. Data on patient information, treatment effects, adverse effects and breast appearance were collected. Network pharmacology was used to identify the effective active ingredients and target information of CHQGT. The Gene Cards database was used to obtain the relevant targets of IGM, and the drug-component–common target relationship network was constructed using Cytoscape 3.9.1 software.

**Results:** All treatment groups showed significant differences in Visual Analog Scale score, Hamilton Depression Rating Scale score, Hamilton Anxiety Rating Scale, mass size and the total effective rate (*p* < 0.001). There was a statistically significant difference in the rate of excellent breast shape between the three groups after treatment (*p* < 0.001), with the rate higher in the CHQGT liver decoction combined with glucocorticoids treatment group compared with the control group. There was a statistically significant difference in the incidence of adverse reactions and recurrence rate between the three groups within 2 years after treatment (*p* < 0.001), with the incidence of adverse reactions and recurrence rate higher in the control group than in the glucocorticoid combination and CHQGT decoction combination groups. Network pharmacology identified 199 active ingredients and 23 drug-disease targets of CHQGT. The molecular docking results showed that the main active components screened had good binding activity with their corresponding target proteins.

**Conclusion:** The combination of CHQGT and MWA is comparable in overall therapeutic efficacy to the combination of glucocorticoids and MWA. However, the CHQGT and MWA combination is superior in reducing lump size, alleviating patient pain and accelerating recovery.

## 1. Introduction

Idiopathic granulomatous mastitis (IGM), also known as granulomatous lobular mastitis, is a rare benign inflammatory disease of the breast, first described by Kessler and Wolloch in 1972 [1, 2]. The histological characteristic of IGM is noncaseating granulomatous inflammation, centered on the breast lobules, with or without microabscesses [3]. The disease typically presents as a unilateral, firm tender mass with sizes ranging from 0.5 to 10 cm, with overlying skin inflammation, while sometimes it may also manifest as a nontender mass or pain alone [4]. Nipple retraction, sinus formation and axillary lymph node enlargement can also be observed [5, 6]. The most common symptoms of IGM reported in the literature are breast mass (48%–100%), fistula or abscess (16%–52%) and inflammation or erythema (11%) [7–12]. The condition usually occurs during the reproductive years, between the ages of 20 and 40, typically within 2 months–15 years after childbirth [13, 14]. The disease is both self-limiting and recurrent [15, 16], with prolonged inflammatory damage and repeated episodes in severe cases resulting in scarring and breast deformation [17], negatively impacting the health and quality of life of women.

While IGM is a rare benign breast disease, its incidence has been rising in recent years, potentially due to increased pathological diagnosis. Studies indicate that IGM accounts for 24% of all inflammatory breast diseases. The annual prevalence rate and incidence rate of IGM are 2.4 per 100,000 women and 0.37%, respectively. Its recurrence rate ranges between 5% and 50%, with a history of pregnancy, breastfeeding, breast infections and smoking established as risk factors for IGM recurrence [18]. Among Han Chinese women with benign breast diseases, IGM constitutes 3.5% [19].

Currently, the exact aetiology and mechanisms underlying IGM remain unclear, and its treatment is controversial. The preferred treatment method for IGM has not yet been standardised. Current treatment options include close monitoring, antibiotic therapy, surgical excision (limited or extensive) and immunosuppressive drugs such as glucocorticoids, methotrexate and azathioprine [20, 21]. Although surgical excision is a common treatment for IGM, studies have shown that it carries a high risk of poor wound healing, fistula or abscess formation and recurrence, with recurrence rates and the incidence of abscesses or fistulas varying from 5% to 50% and 4.7%–30%, respectively [4, 5, 9, 13, 20]. The disease is typically classified as nonlactational mastitis. In addition to surgery, conservative treatments may also involve the use of glucocorticoids and/or triple therapy for tuberculosis, which includes methotrexate and other immunosuppressive drugs. However, due to significant side effects that may occur during treatment, extended treatment durations, recurrent abscesses and persistent wound infections, patients often struggle to complete the entire treatment regimen, potentially leading to the need for surgical intervention [22, 23]. Therefore, developing more effective treatment strategies is crucial for improving the therapeutic outcomes and quality of life for patients with IGM.

In traditional Chinese medicine, this disease is classified under the category of ‘comedomastitis.' It is believed that its onset is often due to nipple deformities or emotional disturbances leading to liver qi stagnation, resulting in blocked meridians and disrupted qi and blood flow. This stagnation transforms into phlegm; stagnant qi and blood can turn hot, causing the flesh to rot and form pus, which after breaking down results in a fistula. If the stagnated qi transforms into fire, it can cause nipple bleeding [24]. Treatment methods include multiple incisions for drainage, curettage, ointment bandaging and the external application of medicinal gauze infused with herbs that promote granulation and remove necrotic tissue. Internal medications aim to invigorate qi, nourish blood, drain pus, detoxify, soften hardness and disperse nodules [25]. This comprehensive treatment yields satisfactory results, benefiting the preservation of the breast's appearance and offering long-term advantages concerning recurrence rates. In ‘Surgery of Traditional Chinese Medicine' [24], for the liver meridian heat type of comedomastitis, the chosen prescription is Chai Hu Qing Gan Tang (CHQGT) (*Bupleurum* liver-clearing decoction), the main ingredients of which include *Bupleurum* (*Chai Hu*), red peony, selfheal and *Hedyotis diffusa*. This formula can clear heat, cool blood, soothe the liver, regulate qi, reduce swelling and disperse nodules [26]. It significantly alleviates the clinical symptoms of granulomatous mastitis, aids recovery, has minimal toxic side effects, a low recurrence rate and certain safety.

Microwave ablation (MWA) under ultrasound guidance is a significant minimally invasive intervention, applied to various benign and malignant tumours. With precise ultrasound guidance, the IGM lesion is punctured, followed by MWA. By adjusting microwave emissions, denatured proteins from the diseased cells are used to remove the affected tissue. The procedure duration for MWA is shorter and can treat larger lesions, alleviating patients' pain. Ultrasound guidance achieves accurate positioning, preventing the missed diagnosis of affected tissues. Moreover, the high temperature leads to the loss of immunogenicity in the affected tissues, eliminating IGM at its root and reducing its recurrence.

Therefore, combining CHQGT with MWA integrates the advantages of both, further enhancing therapeutic effects and shortening the disease course. However, currently, there is limited research on the combined treatment of IGM using CHQGT and MWA. This study focuses on patients with IGM in exploring the clinical effects of this combined treatment approach in their therapeutic process. The possible pharmacological mechanism is analysed by means of network pharmacology.

## 2. Research Participants and Methods

### 2.1. Clinical Research Section

#### 2.1.1. Research Participants

Using convenience sampling based on age, we retrospectively analysed 480 cases of patients with IGM treated in the Breast Surgery Department of our hospital between January 2020 and December 2021. All patients were diagnosed with IGM by core needle biopsy prior to hospitalisation. The random number table method was used to group the patients according to the treatment method. The operation steps were as follows: (1) the patients were numbered by drawing lots; (2) starting from any number in the random number table, a random number was obtained for each experimental unit in the same direction; and (3) for grouping, the random numbers were arranged from small to large (if the numbers were the same, the first number was placed in front). The patients corresponding to the first 160 random numbers were included in the CHQGT combination group (CHQGT + MWA), the patients corresponding to the middle 160 random numbers were included in the glucocorticoid combined group (glucocorticoids + MVA) and the last 160 patients were included in the control group (glucocorticoids).

**Figure 1:**
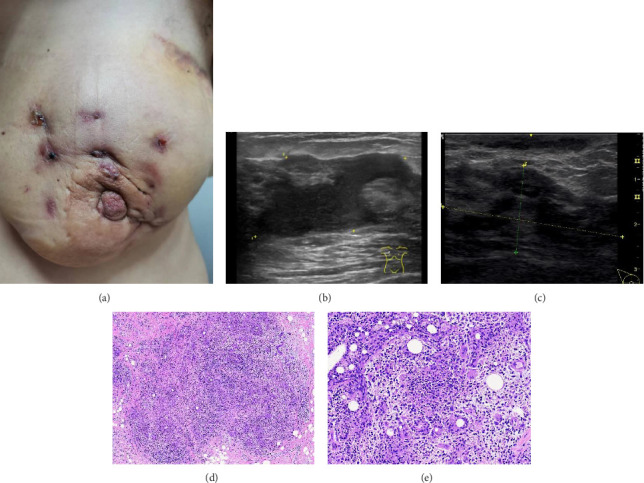
Diagnosis of severe IGM. (a) The breast appearance of a typical case with severe IGM. (b) Breast ultrasonography of a large lesion with abscess formation. (c) Breast ultrasonography of fused multiple lesions. (d) Pathology magnification ×100. (e) Pathology magnification ×400.

IGM was diagnosed by combining the patient's clinical manifestations, physical examination, breast ultrasonography and histopathological findings (Figure 1). Most of the clinical manifestations of these patients were painful masses on one side of the breast, and there was no obvious abnormality in local skin colour and temperature at the beginning of the disease. As the disease progresses, more than 50% of patients have inflammatory changes in the affected side of the breast, such as erythema and swelling. Other symptoms include sunken nipples, fistulas and ulcers, and around 37% of patients develop abscesses. Lesions can occur in any quadrant of the breast, mostly from the peripheral quadrant, gradually spreading to the areola area and even to the entire breast in a short time. Some patients may be accompanied by fever, chills, rash and other systemic symptoms [27]. The typical ultrasonographic manifestations of IGM are multiple adjacent hypoechoic masses with posterior acoustic shadow or posterior acoustic shadow enhancement. Advanced cases show effusion, cavity or even strip hypoechoic (sinus); rich blood flow signals or signs of angiogenesis can be seen in the diseased area, and 15%–55% of cases can exhibit ipsilateral axillary reactive lymph node enlargement [28]. The final diagnosis of IGM depends on histopathology, and hollow needle biopsy is the first choice of method. The disease's typical pathological feature is the formation of a noncaseous necrotic granuloma with breast lobule as the centre, accompanied by local infiltration of multinucleated giant cells, epithelioid cells, lymphocytes and plasma cells [29]. In traditional Chinese medicine, IGM belongs to the category of ‘acne mastitis' (female nipple belongs to ‘liver' and breast belongs to ‘stomach'), which is characterised by loss of liver qi and stagnation of qi. Stagnation of liver qi leads to obstruction of milk collaterals, stagnation of liver and stomach, phlegm and blood stasis, obstruction of breast collaterals, agglomeration, heat for long periods and meat rot resulting in abscess and fistula following ulceration [30].

This study was approved by the ethics committee of our hospital (Ethics No. 202044). All participants were informed and signed written consent forms. The inclusion criteria were as follows: patients with (1) signs of liver-qi depression, such as breast pain, dysmenorrhea chest tightness, ventosity, constipation and depression; (2) onset within 1 month, without undergoing hormone, antituberculosis drug, immunotherapy or surgical treatment (excluding simple abscess drainage surgery), and those requiring conservative drug treatment during the acute progression period; and (3) women aged between 18 and 60 years old (inclusive of both 18 and 60).

The exclusion criteria included (1) coexistence of malignant breast tumours, other endocrine diseases, autoimmune diseases or inflammatory diseases with symptoms similar or identical to those of IGM; (2) coexistence of other breast diseases, or patients taking medication that might influence the study results; (3) coexistence of severe cardiovascular diseases, primary diseases such as liver or kidney dysfunction, or mental disorders; (4) pregnant or lactating women; and (5) individuals with a predisposition to allergies or known allergies to the class of drugs or its components.

The elimination criteria were as follows: (1) pregnancy during treatment; (2) poor compliance, failing to attend follow-up visits or provide relevant case report information; and (3) discovery of other violations of the inclusion criteria or meeting the exclusion criteria during the study.

The termination criteria included (1) serious adverse reactions during the trial (mainly adverse reactions to the use of glucocorticoids, including osteoporosis, osteonecrosis, aggravated infection, induced or aggravated gastric and duodenal ulcers); (2) unexpected circumstances arose, making it challenging to evaluate the drug's efficacy; and (3) treatment was forcibly terminated due to other diseases (mainly secondary diabetes and other diseases that could not be addressed in this study).

#### 2.1.2. Research Methods

For the control group, prednisone was used for treatment, starting at a dose of 20 mg/d. Upon symptom relief, the dosage was gradually reduced every 1-2 weeks sequentially to 16, 12, 8, and 4 mg/d, and was maintained at 4 mg/d until the end of the treatment course. The judgment was made by clinicians based on expert consensus. The criteria for determining symptom relief are the disappearance of clinical symptoms, the inaccessibility of the original inflammatory lesions clinically and the healing of ulcers or sores, but the presence of scattered minor lesions is still visible on imaging [31]. The total duration of treatment was 3 months. Patients unresponsive to the treatment underwent surgical excision. The criteria for determining such patients are as follows: (1) clinical symptoms (breast lump size, redness, swelling and pain) are not relieved after 1 week of treatment; (2) imaging shows no response to treatment (stable or enlarged lesion range); and (3) the granulomatous lobular mastitis disease activity index remains unchanged or increases [31].

For the glucocorticoids combined group, MWA treatment was added to the regimen of the control group. The MWA device (KY-2000, Nanjing Canyon Medical Technology Co., Nanjing, China) operated at a frequency of 2450 MHz. The MWA needle had an outer diameter of 16G and a transmission tip length of 3 mm. Ultrasound examinations were performed using the American GE Logiq P9 ultrasound device (probe: 9–12 LMHz) and operated on an ultrasound imaging software platform equipped with an Italian Demetro 16G semiautomatic biopsy needle. Ultrasound was used to determine the lesion's location, size, number, blood flow signals and echo characteristics. Local infiltration anaesthesia was administered to the patient's breast skin and retromammary space prior to treatment. Physiological saline was injected into lesion areas close to the skin, areola and muscles to form a barrier to prevent thermal injury. Individualised ablation treatment plans were formulated based on ultrasound results of the lesion. The treatment plan included determining the needle insertion point, needle insertion depth, number of needle insertions, lesion fluid aspiration and duration of ablation. The microwave power was set to 25–30 W. Under ultrasound guidance, the active tip of the MWA needle was placed within the lesion, and the entire process was continuously observed under dynamic ultrasound in multiple sections. Dilated milk ducts were gradually ablated from deep to shallow along the duct wall. Micro-abscesses were treated with pinpoint ablation, and multipoint, multilevel mobile ablation was used for multiple lesions and larger lesions (maximum diameter: > 2 cm). Contrast-enhanced ultrasound was employed to determine whether the ablation was complete (intraoperative ultrasound monitoring with SonoVue infusion into the cubital vein [2.4 mL]). Ablation was considered complete when no contrast agent perfusion was observed in the ablation area. If the ablation was incomplete, the ablation process had to be repeated immediately. Following ablation, patients were treated with local dressings and ice packs and were discharged 4–6 h later. Patients unresponsive to the treatment underwent surgical excision.

For the CHQGT combination group, treatment was administered using CHQGT combined with MWA, with the MWA procedure being the same as described above. The prescription for CHQGT is as follows: 9 g of *Bai Gui* (*Paeonia lactiflora*), 9 g of *Dang Gui* (*Angelica sinensis*), 15 g of *Bai Jian* (*ampelopsis japonica*), 10 g of rhizome, 10 g of turmeric, 6 g of dried citrus peel, 5 g of licorice, 18 g of dandelion and 18 g of *Shi Zi* (*Ardisia chinensis* Benth). Following preparation, the patient took the decoction orally once a day, and continued this for a month, until the lesion completely disappeared or the treatment was ineffective. Patients unresponsive to the treatment underwent surgical excision.

#### 2.1.3. Data Collection

We aimed to compare the overall treatment effect of the three groups, including the effect on reducing lump size, reducing patient pain and accelerating recovery. Data regarding the patient's age, body mass index (BMI), number of lesions, size of lesions, distribution of lesions, marital and childbirth history, white blood cell count (WBC), percentage of neutrophils (NEUT%), C-reactive protein (CRP), efficacy at the end of the study and breast appearance following treatment were collected. After treatment, data from 1, 3 and 5 months were collected, including Visual Analog Scale (VAS), Hamilton Depression Rating Scale (HAMD) and Hamilton Anxiety Rating Scale (HAMA) scores, and lump size. After the end of treatment, the patients were followed up for 2 years, and the patients were requested to undergo a follow-up every 3 months to investigate any recurrence and adverse reactions.

In terms of efficacy, with reference to the *Standards of Diagnosis and Therapeutic Effect of TCM Diseases and Syndromes* from 1994 in the People's Republic of China, the outcomes were classified into the following: (1) cured: breast redness, swelling, heat and pain had disappeared, the fistula healed, no pseudo-healing and all systemic symptoms had disappeared; (2) improved: most of the lump had disappeared or most of the fistula had healed, with superficial wounds remaining unhealed; and (3) ineffective: the breast still exhibited redness, swelling, heat and pain, the fistula did not heal, or the lesion area had even expanded. The efficacy rate = (cured + improved)/*N* × 100% [32].

Based on the standards jointly established by the American Surgical Association, Radiological Society, Pathological Society and the Surgical Oncology Society, breast appearance was categorised as follows: (1) excellent: the size and shape of the breast after treatment are roughly the same as the contralateral breast; (2) good: retraction and skin changes of the breast involve less than 1/4 of the original; (3) fair: retraction and skin changes involve between 1/4 and 1/2 of the breast; (4) poor: breast deformity involves more than 1/2 of the breast. The good-to-excellent rate = (excellent + good)/total cases × 100% [33].

The VAS score was used to evaluate the pain situation before and after treatment in the three groups, with the score directly proportional to the level of pain. A score of 0 indicates no pain, with a maximum of 10. Scores of < 3 indicate mild pain, 4–6 represents moderate pain and > 7 indicates severe pain [34].

The HAMD was developed by Hamilton in 1960. The scale has 24 items across seven categories: anxiety/somatisation, weight, cognitive impairment, diurnal variation, retardation, sleep disturbances and feelings of despair. In terms of scoring criteria, a total score of > 35 indicates severe depression, 21–35 signifies mild-to-moderate depression, 8–20 represents borderline depression and < 8 means no depression [35].

The HAMA is used to evaluate patients anxiety symptoms before and after nursing. A score of < 7 indicates no anxiety symptoms and is psychologically normal; scores of > 7 but < 14 suggest possible anxiety symptoms; scores of > 14 but < 21 indicate definite anxiety symptoms; scores of > 21 but < 29 confirm significant anxiety symptoms; and scores of > 29 represent severe anxiety symptoms. The score is inversely proportional to the severity of anxiety symptoms; the lower the score is, the milder the symptoms [36].

#### 2.1.4. Statistical Analysis

Statistical analysis was conducted using SPSS 26.0 software. Data conforming to a normal distribution were expressed in terms of means and standard deviation (*x* ± *s*). Intergroup comparisons were performed using analysis of variance (ANOVA), with pairwise comparisons utilising the least significant difference method. Count data were expressed as frequency and percentage, with the chi-square (*χ*^2^) test or Fisher's exact test used for intergroup comparisons and the chi-square partition method for pairwise comparisons. A significance level of *p* < 0.05 indicated statistical significance.

### 2.2. Network Pharmacology Section

#### 2.2.1. Screening of Active Ingredients and Related Targets of Bupleurum Qinggan Decoction

In the traditional Chinese medicine systems pharmacology database and analysis platform (TCMSP) (https://tcmspw.com/tcmsp.php/), the effective active ingredients of the formula were screened using 12 traditional Chinese medicines of Bupleurum Qinggan *Tang* as keywords, with oral bioavailability ≥ 30% and drug likeness ≥ 0.18 used as the limiting conditions. The target protein was converted into a standard name using the UniProt database (https://sparql.uniprot.org/).

#### 2.2.2. Acquisition of IGM-Related Targets and Key Targets

The keyword ‘idiopathic granulomatous mastitis' was searched in the Gene Cards (https://www.genecards.org/) database to obtain disease-related targets. The drug active ingredient targets and disease targets were introduced into Venny 2.1.0 to create a Wayne diagram, and the drug/disease key targets of the CHQGT decoction for the treatment of IGM were obtained.

#### 2.2.3. Construction of Chai Hu Qinggan Decoction for the Treatment of IGM Target Network

The data on the drugs, active ingredients, disease-related targets and their attribute files were imported into Cytoscape 3.9.1, and the composition–disease–target network diagram of Bupleurum Qinggan decoction and IGM was plotted.

#### 2.2.4. Protein–Protein Interaction (PPI) Network Construction of Intersecting Targets

The drug–disease intersection target was input into the STRING database (https://www.string-db.org). The species was selected as ‘Homo sapiens,' the minimum interaction threshold was set to ‘medium confidence (0.4)' and the PPI network was obtained. The results were imported into Cytoscape 3.9.1 for visualisation.

#### 2.2.5. Gene Oncology (GO) Enrichment and Kyoto Encyclopedia of Genes and Genomes (KEGG) Pathway Enrichment Analysis

The intersection targets of the CHQGT decoction in the treatment of IGM were imported into the DAVID database (https://david.ncifcrf.gov/), and *p* < 0.05 was set. GO enrichment analysis was performed in terms of three modules: biological process (BP), cellular component (CC) and molecular function (MF). The entries of the enrichment results were plotted as bar charts and bubble charts using an online data analysis visualisation platform (https://www.bioinformatics.com.cn/).

#### 2.2.6. Molecular Docking Verification of Key Targets

The top 5 core active ingredients were calculated according to the topological parameter Degree value, and the three-dimensional (3D) structure in Mol2 format was downloaded from the TCMSP database and the structure was imported into ChemBio3DUltra 14.0 for energy minimisation. The 3D structure of the core protein gene was downloaded from the Protein Data Bank database, and Pymol2.3.0 was used to remove protein crystal water and original ligands. AutoDock was used (v1.5.6) to hydrogenate the core active ingredients and key targets, calculate the charge, etc. and save them in PDBQT format. Then, POCASA 1.1 was used to predict the protein binding site and AutoDock Vina1.1.2 was used for docking. The PyMOL 2.3.0 and LigPlot V2.2.5 systems were used to analyse the interaction mode of the docking results. The binding activity of the compound and the core protein target was evaluated according to the binding energy, with the binding energy of < −5.0 kJ/mol selected as the screening condition to evaluate the docking effect of the two.

## 3. Results

### 3.1. Clinical Research Part

#### 3.1.1. General Information

The results indicated that before treatment, there were no statistically significant differences among the three groups of patients in terms of age, BMI, smoking history, alcohol consumption history, number of lesions, size of lesions, distribution of lesions, marital and fertility history, WBC, NEUT% and CRP (all *p* > 0.05). This suggested that the three groups of patients were comparable, as shown in Table 1.

#### 3.1.2. Changes in Patients' Lump Size and Psychological Status After Treatment

**Figure 2:**
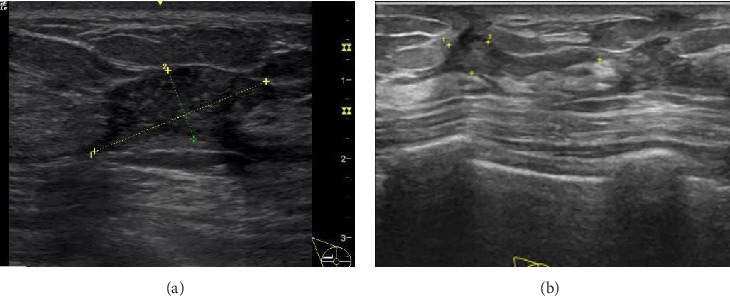
Treatment outcomes of the enrolled patients. (a) Lesion volume detection under ultrasonography for a typical case at 3 months after MWA. (b) Lesion volume detection for a typical case under ultrasonography at 5 months after MWA.

Colour Doppler ultrasound was used to monitor changes in lesion volume during treatment (Figures 2(a) and 2(b)). Single-factor repeated-measures ANOVA was employed to investigate the effects of different treatment methods on VAS scores, HAMD scores, HAMA scores and lump sizes for the three patient groups. According to the Shapiro–Wilk test, the data of each group followed an approximate normal distribution (*p* > 0.05). According to Mauchly's test of sphericity, the variance–covariance matrices of the groups were equal (*p* > 0.05). Data were presented as *x* ± *s*, as shown in Table 2. The results are summarised as follows. The interaction of time ∗ treatment was significant in VAS scores, HAMD scores, HAMA scores and lump sizes across the three groups (*F*_VAS_ score = 322.111, *p* < 0.001; *F*_HAMD_*score* = 219.432, *p* < 0.001; *F*_HAMA_*score* = 112.122, *p* < 0.001; *F*_lump⁣size_ = 144.561, *p* < 0.001), indicating that the individual effect changes in VAS scores, HAMD scores, HAMA scores and lump sizes were different among the three groups. Moreover, with the extension of time, the scores and lump sizes in all three groups generally decreased (*F*_VAS_ score = 123.456, *p* < 0.001; *F*_HAMD_ score = 245.423, *p* < 0.001; *F*_HAMA_ score = 247.218, *p* < 0.001; *F*_lump⁣size_ = 145.131, *p* < 0.001), indicating a significant change in the patients' VAS scores, HAMD scores, HAMA scores and lump sizes over time. Lastly, the influence of different treatment methods on VAS scores, HAMD scores, HAMA scores and lump sizes varied across the three patient groups (*F*_VAS_ score = 145.423, *p* < 0.001; *F*_HAMD_ score = 144.393, *p* < 0.001; *F*_HAMA_ score = 145.642, *p* < 0.001; *F*_lump⁣size_ = 287.328, *p* < 0.001).

Further pairwise comparisons were made in terms of VAS scores, HAMD scores, HAMA scores and lump sizes across the three patient groups at different time points. There were no statistically significant differences among the three groups in any of the metrics (all *p* > 0.05), suggesting good comparability across the groups. The descriptions of the metrics for the three groups at 1, 3, and 5 months are as follows. For VAS scores, at 1 and 3 months, the ranking was CHQGT combination group < corticosteroid combination group < control group; at 5 months, CHQGT combination group = corticosteroid combination group < control group. For HAMD and HAMA scores, the ranking at 1, 3, and 5 months were the same, with CHQGT combination group = corticosteroid combination group < control group. For lump size, at the first month, the ranking was CHQGT combination group = corticosteroid combination group < control group; at 3 and 5 months, it was CHQGT combination group < corticosteroid combination group < control group (Table 2).

#### 3.1.3. Comparison of Treatment Efficacy Among the Three Groups

The overall effective rates were 91.88%, 87.50% and 67.50% in the CHQGT combination group, glucocorticoids combination group and control group, respectively. The comparison results of treatment efficacy showed a statistically significant difference in the overall effective rate among the three groups (*χ*^2^ = 55.824, *p* < 0.001). Pairwise comparison revealed that the CHQGT combination group and glucocorticoids combination group had a better curative effect than the control group. However, there were no significant difference between the CHQGT combination group and the glucocorticoids combination group. In addition, the disease duration of the CHQGT, glucocorticoids and control groups were compared, with the durations 5.54 ± 3.03, 5.85 ± 3.04, 8.24 ± 3.21 months, respectively. There was a significant difference in the disease duration among the three groups (*F* = 11.360, *p* < 0.001). Following pairwise comparison, the difference of disease duration among the groups was statistically significant (Table 3).

#### 3.1.4. Comparison of Adverse Reactions During Treatment

Within 2 years after treatment, 4 patients in the CHQGT combination group, 15 patients in the corticosteroid combination group and 34 patients in the control group experienced adverse reactions, with overall adverse reactions incidence rates of 2.50%, 9.38% and 21.25%, respectively, showing a statistical difference among the three groups (*p* < 0.001). The comparison of adverse reactions showed that the incidence rate of adverse reactions during treatment in the CHQGT combination group and glucocorticoids combination group was significantly lower than that in the control group (Table 4).

#### 3.1.5. Comparison of Recurrence

The results showed that within 2 years after treatment, 30 patients in the combination group, 35 in the glucocorticoids group and 50 in the control group were readmitted to the hospital for relapse, with recurrence rates of 18.75%, 21.88% and 31.25%, respectively. The difference in recurrence rates between the three groups was statistically significant (*χ*^2^ = 7.433, *p*=0.024). Among them, the recurrence rate of the combination group and the glucocorticoids group was lower than that of the control group (*p* < 0.05), whereas there was no significant difference in the recurrence rate between the combination group and the glucocorticoids group (*p* > 0.05).

#### 3.1.6. Comparison of Breast Appearance After Treatment

**Figure 3:**
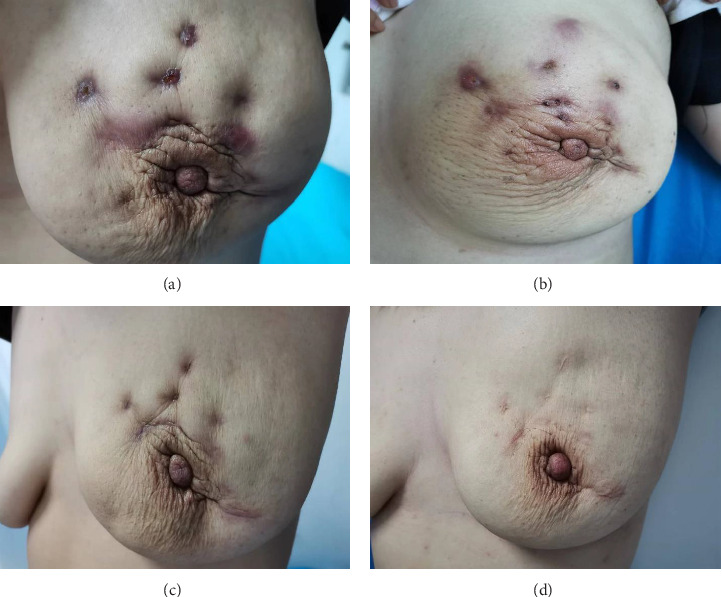
The alteration of breast appearance after the therapy in a typical case with severe IGM. (a) 2 weeks after MWA; (b) 1 month after MWA; (c) 3 months after MWA; (d) 5 months after MWA.

The comparison results of breast appearance post-treatment showed statistically significant differences in the good-to-excellent rate of breast appearance among the three groups (*χ*^2^ = 45.366, *p* < 0.001), with 84.38% in the CHQGT combination group, 64.38% in the glucocorticoids combination group and 48.75% in the control group. Pairwise comparisons indicated that the good-to-excellent rate of breast appearance in the CHQGT combination group and glucocorticoids combination group was significantly higher than that in the control group (Table 5). Changes in the typical appearance of the breast after treatment are shown in Figure 3.

### 3.2. Network Pharmacology Section

#### 3.2.1. Target Network Analysis of Bupleurum Qinggan Decoction and IGM

**Figure 4:**
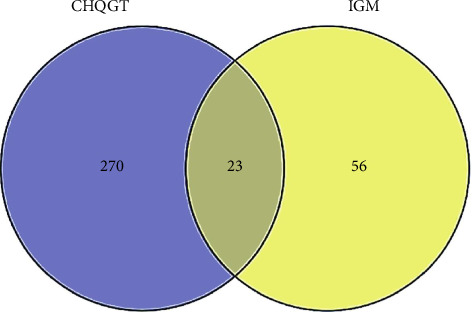
Wayne diagram of the intersection of the active ingredient of CHQGT and IGM.

**Figure 5:**
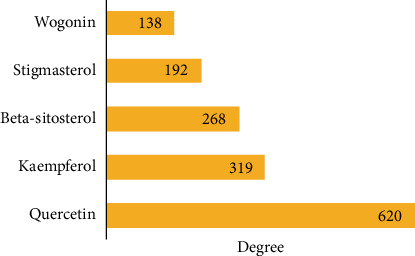
Top 5 CHQGT active ingredients.

A total of 199 active ingredients, 293 active ingredient targets and 79 IGM disease-related targets were obtained after screening, of which 23 were active ingredient and disease intersection targets (Figure 4). A total of 478 nodes and 4374 edges were obtained using Cytoscape 3.9.1 to construct the network of Bupleurum Qinggan decoction and IGM, and Network Analyser was used to analyse the topological parameters of PPI. Ranked by Degree value, the top 5 active pharmaceutical ingredients were quercetin (MOL000098, quercetin), kaempferol (MOL000422), *β*-sitosterol (MOL000358, beta-sitosterol), stigmasterol (MOL000449) and baicalein (MOL000173, wogonin) (Figure 5). It is speculated that the Bupleurum Qinggan decoction may play a key role in the treatment of IGM through the above active ingredients.

#### 3.2.2. Analysis of Core Targets of Bupleurum Qinggan Decoction in the Treatment of IGM and Construction of PPI Network

**Figure 6:**
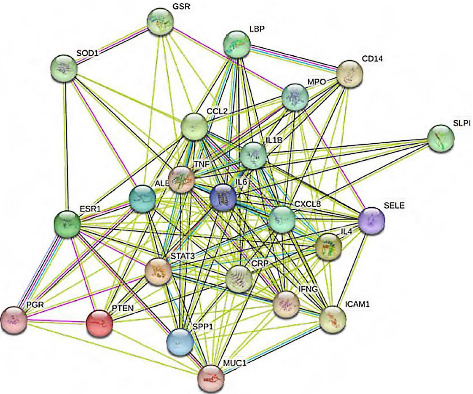
PPI network diagram of a key target of IGM in the treatment of CHQGT.

**Figure 7:**
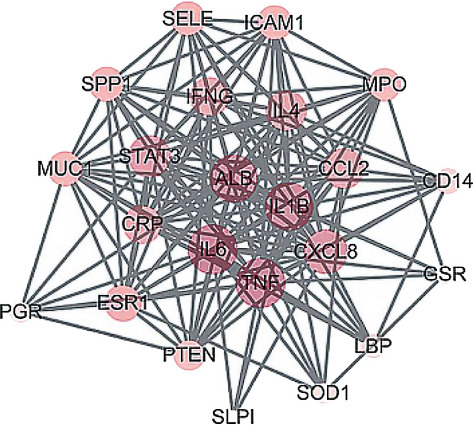
Core targets of CHQGT in the treatment of IGM.

A total of 23 intersecting targets were imported into the STRING database for PPI network analysis, and 23 nodes and 163 edges were obtained after screening (Figure 6). The results were imported into Cytoscape 3.9.1, and the PPI topological parameters were analysed using Network Analyzer. The top 10 nodes were selected as the core targets, namely interleukin (IL)-6, tumour necrosis factor (TNF), albumin, IL-1β, C-X-C motif chemokine ligand 8 (CXCL8), signal transducer and activator of transcription 3 (STAT3), C-C motif chemokine ligand 2, IL-4, interferon gamma and CRP, and the visual network diagram was created (Figure 7). It is speculated that the Bupleurum Qinggan decoction acts on the above targets and plays a role in the treatment of IGM.

#### 3.2.3. GO Enrichment Analysis and KEGG Pathway Enrichment Analysis

**Figure 8:**
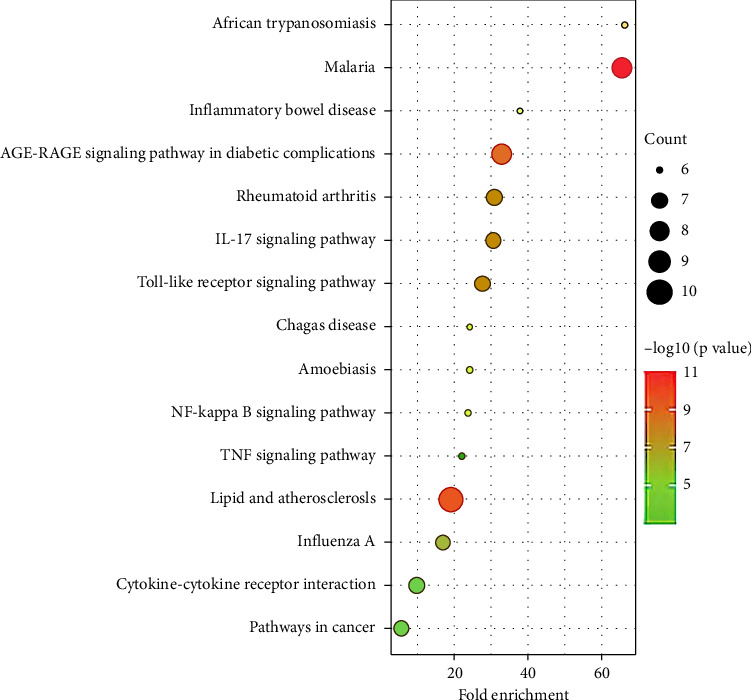
KEGG pathway enrichment map of CHQGT treatment of IGM intersection targets.

A total of 23 drug-disease intersection targets were imported into the DAVID database for GO enrichment analysis, and *p* < 0.05 was used as the screening condition, with a total of 129 items for BP enrichment, 9 items for CC enrichment and 13 items for MF enrichment. The results showed that BP mainly included inflammatory response, positive regulation of gene expression, cellular response to lipopolysaccharide and positive regulation of DNA transcription. Moreover, CC mainly includes extracellular space, extracellular region, exosomes and plasma membrane, and MF mainly includes protein binding, identical protein binding, cytokine activity, enzyme binding and receptor binding. We constructed a KEGG functional enrichment map based on the count of disease-related genes or proteins (Figure 8), which primarily included 15 core signaling pathways such as IL-17, toll-like receptors, cytokine receptor interactions, nuclear factor kappa B (NF-κB), and TNF.

#### 3.2.4. Connection Between the Main Active Ingredients of Bupleurum Qinggan Decoction and the Molecules of Key Targets

According to the results of network pharmacology analysis, the top 5 active ingredients with the highest degree value were quercetin (MOL000098), kaempferol (MOL000422), *β*-sitosterol (MOL000358, beta-sitosterol), stigmasterol (MOL000449) and baicalein (MOL000173, wogonin), along with IL-6 (PDBID: 1ALU), TNF (PDBID: 2E7A), IL-1β (PDBID: 6I8Y), CXCL8 (PDBID: 6N2U), STAT3 (PDBID: 6NJ2) and IL-4 (PDBID: 2D48). The binding energy of the key active ingredients and the core target was ≤ −5.0 kJ/mol.

## 4. Discussion

Corticosteroid treatment is considered the primary treatment for IGM. Prednisone, in particular, is a commonly used medication for the clinical management of IGM and belongs to the corticosteroid class. It can control the expression of cell adhesion molecules and, once inside the body, can transform into potent metabolic products. These properties allow it to prevent the adhesion of inflammatory cells and exert anti-inflammatory effects [37]. Prednisone can also influence the activity of arginase and NF-κB, inhibiting the maturation and differentiation of peripheral dendritic cells, obstructing signal transmission and nuclear transcription in lymphocytes and controlling lymphocyte-mediated immune responses [38]. Using prednisone to treat IGM can effectively alleviate local inflammatory reactions in the breast [39]. In a randomised clinical trial, 30 patients with IGM underwent 2 months of prednisone treatment at different doses. It was found that the remission rate for the high-dose group (starting dose of 50 mg, halving the dose every 3 days to the same 5-mg dose as the low-dose group) was significantly higher than that for the low-dose group (93.3% vs. 53.3%, *p*=0.03). Among those who experienced remission, the relapse rate in the high-dose group was also significantly reduced (0% vs. 37.5%, *p*=0.04). This suggests that high-dose prednisone treatment for IGM has a higher success rate, a lower recurrence rate and reduces the need for surgery [40].

Through precise ultrasound-guided puncture of the lesion, MWA adjusts the effective thermal field emitted by microwaves. Utilising the high temperatures generated by oscillations of water molecules within tissues, it can directly coagulate and denature the proteins of lesion cells, leading to the complete necrosis of the lesion tissue. Advantages of this method include sustained high temperatures, substantial tumour ablation, short operation time and low pain scores. Under ultrasound guidance, precise localisation can be achieved without missing the lesion. As the proteins of the lesion tissue denature at high temperatures, the immunogenicity of the lesion tissue is lost. Therefore, factors leading to IGM recurrence can be fundamentally eliminated. Thus, the combined use of MWA with glucocorticoids can further enhance the therapeutic effect and promote recovery. However, it is worth noting that prednisone has a series of adverse reactions, such as allergic reactions, weight gain, diarrhoea, lower limb edema and menstrual irregularities, which can affect treatment compliance to some extent.

In ‘Surgery of Traditional Chinese Medicine' [24], the medication prescribed for acne-like breast abscess of the liver-heat type is CHQGT. This decoction can significantly improve the clinical symptoms of granulomatous mastitis, promote recovery, has minimal toxic side effects and a low relapse rate, and is relatively safe. Recent pharmacological research has shown that the effective components of *Bupleurum* are mainly saponins, flavonoids and lignans. *Bupleurum* (*Chai Hu*) can inhibit the release of inflammatory mediators, the growth of granulomas and antiexudation, as well as suppress the elevating capillary permeability caused by histamine and 5-hydroxytryptamine. It also significantly inhibits other inflammatory responses, such as leukocyte migration and connective tissue proliferation [41]. Recent pharmacological studies indicate that *Bupleurum* has immunomodulatory effects, which may be realised through the regulation of the PI3K/AKT/NF-κB/NLRP3 pathway [42]. Flavonoid compounds are the main active ingredients in *Scutellaria*, exhibiting antioxidant, anti-inflammatory and antitumour properties, with inhibitory effects on both acute and chronic inflammation [43]. Yang Jiangping [44] stated that the combination of *Bupleurum* and *Scutellaria* has a synergistic effect, enhancing antipyretic results. This might be related to the regulation of central body temperature, adjusting prostaglandin E2 and cAMP, and inhibiting endogenous pyrogens TNF-α and beta-endorphin synthesis or release. *Forsythia* possesses anti-inflammatory, antibacterial, antiviral, antioxidant, hepatoprotective and antitumour actions [45]. Through observing the antibacterial effects of multiple herbal decoctions on *Acinetobacter baumannii* in vitro, Ye Guanlong [46] discovered that *Forsythia* inhibits the bacterium in vitro. Burdock seeds exhibit antioxidant properties and play roles in anti-inflammation, antibacterial activity and pain relief. Their effective ingredients, coumaric acid and chlorogenic acid, show potent antibacterial activities, especially noteworthy for their inhibitory effects on *Shigella dysenteriae* [47]. Trichosanthin primarily contains polysaccharides, proteins, saponins, amino acids and other effective components. It displays a range of pharmacological activities, including antitumour, anti-inflammatory, antiviral and antibacterial effects. The polysaccharides in Trichosanthin can promote the secretion of TNF-α and IL-6, significantly enhancing immune activity [48]. The main effective ingredient in *Gardenia*, geniposide, can restore the dynamic balance of pro-inflammatory/anti-inflammatory cytokines, regulate immune cell function and activation, reduce the production of inflammatory mediators and inflammatory factors, and adjust various signalling pathways to exert anti-infection and immune-regulating effects [49]. *Saposhnikovia divaricata* (commonly known as *Fangfeng*) possesses pharmacological activities such as antipyretic, anti-inflammatory, analgesic and antitumour effects.

In this study, there was no statistically significant difference in therapeutic efficacy between the CHQGT combination group and the glucocorticoids combined group. However, the former had a shorter duration of disease. This suggests that the combination of CHQGT and MWA can effectively reduce toxic side effects experienced by patients during treatment while improving the condition of the breast shape. In the VAS score comparison, the CHQGT combination group had a lower score than the glucocorticoids combined group at 1 and 3 months, with no difference at 5 months, indicating that the combination can more rapidly alleviate patient discomfort in the early stages of treatment. At 3 and 5 months, the lump size reduction rate in the CHQGT combination group was higher than that in the glucocorticoids combined group.

At present, the main nonsurgical treatment of IGM is steroid hormone therapy, which has good anti-inflammatory, antiallergic and immunosuppressive effects, and can play a role in reducing the lump and the pain when used rationally. However, the dosage and duration of glucocorticoids remain controversial, and the adverse reactions caused by the use of glucocorticoids, such as Cushing's syndrome (central obesity, moon face, hirsutism, etc.), glucose metabolism disorders, abnormal blood pressure and peptic ulcers, mean patients often have poor compliance. On the basis of this study, we can promote the CHQGT treatment model in patients with IGM, which can shorten the length of hospital stay and reduce adverse effects.

This study has certain limitations. First, being a single-centre study, it was challenging to ensure consistent baselines when comparing cohorts, and patients may have had other comorbidities that affected the prognosis. At the same time, due to the limitation of funding and other factors, this study could only adopt the convenience sampling method, and although the relevant baseline data of the enrolled patients were compared, it was still difficult to eliminate the influence of potential confounding factors. As such, multistage stratified random sampling will be used in the follow-up study to conduct relevant research. Second, due to the inconsistent forms of medications, the researchers had to further examine and inquire about the patients' specific medication intake; this study does not adhere to the double-blind principle. Third, for medical ethical reasons and patient medical needs, the enrolled participants were all patients who required drug intervention, making it impossible to establish a blank control group. Last, there is no unified consensus on the diagnosis and treatment of this disease; it remains a contentious point among breast specialists in various regions and a challenge in clinical treatment. High-level evidence supporting different treatment methods is lacking, warranting more in-depth, comprehensive research to devise optimal strategies and establish effective treatment guidelines.

In summary, the combination of CHQGT and MWA is comparable to glucocorticoids combined with MWA in overall efficacy. The CHQGT treatment is superior in terms of reducing tumours, reducing patient pain and accelerating recovery.

## Figures and Tables

**Table 1:** Comparison of general data of three patient groups.

Item	CHQGT combination group (*n* = 160)	Glucocorticoid combination group (*n* = 160)	Control group (*n* = 160)	*χ* ^2^/*F* value	*p* value
Age (years, *x* ± *s*)	32.43 ± 5.23	34.73 ± 5.47	33.25 ± 6.78	2.531	0.084
BMI (kg/m^2^, *x* ± *s*)	22.55 ± 2.41	22.65 ± 1.75	22.46 ± 1.88	0.360	0.700
Smoking history (cases)	18	27	24	2.133	0.344
Alcohol consumption history (cases)	30	33	27	0.738	0.691
Number of lesions (*n*, *x* ± *s*)	3.82 ± 1.40	3.84 ± 1.56	4.43 ± 1.52	2.061	0.135
Size of lesions (mm, *x* ± *s*)	44.68 ± 15.23	47.54 ± 18.40	44.21 ± 12.10	0.634	0.530
Distribution of lesions (left/right)	92/68	90/70	96/64	0.479	0.787
Marital and childbirth history (cases)	160	160	160	—	—
WBC	5.34 ± 1.47	5.24 ± 1.35	5.31 ± 1.20	0.062	0.940
NEUT%	61.54 ± 5.31	59.52 ± 5.53	61.67 ± 6.42	1.055	0.355
CRP	5.65 ± 2.43	4.65 ± 1.81	4.62 ± 1.42	1.062	0.364

*Note:* NEUT% = neutrophil percentage, WBC = white blood cell count.

Abbreviation: CRP = C-reactive protein.

**Table 2:** Changes in patient's lump size and psychological condition after treatment.

Item		CHQGT combination group (*n* = 160)	Glucocorticoid combination group (*n* = 160)	Control group (*n* = 160)	*F*_interaction_/*P*_interaction_ value	*F*_time_/*P*_time_ value	*F*_treatment_/*P*_treatment_ value
VAS	Baseline^b^	6.43 ± 1.22	6.41 ± 1.50	6.32 ± 1.67	322.111/< 0.001	123.456/< 0.001	145.423/< 0.001
	1 M^a^	3.65 ± 1.24	4.26 ± 1.25	5.23 ± 1.59			
	3 M^a^	2.26 ± 0.84	3.21 ± 1.40	4.31 ± 1.26			
	5 M^a^	1.33 ± 0.78	1.54 ± 0.74	3.42 ± 1.32			

HAMD	Baseline^b^	25.50 ± 3.54	24.42 ± 3.51	26.54 ± 3.82	219.432/< 0.001	245.423/< 0.001	144.393/< 0.001
	1 M^a^	19.78 ± 3.67	20.58 ± 3.52	23.54 ± 3.68			
	3 M^a^	12.34 ± 2.67	13.61 ± 3.76	18.77 ± 1.76			
	5 M^a^	7.61 ± 2.33	7.43 ± 2.16	11.51 ± 2.13			

HAMA	Baseline^b^	26.67 ± 5.52	27.42 ± 5.45	27.34 ± 4.14	112.122/< 0.001	247.218/< 0.001	145.642/< 0.001
	1 M^a^	21.56 ± 4.55	22.57 ± 4.52	24.45 ± 3.68			
	3 M^a^	14.23 ± 3.34	15.43 ± 4.34	17.42 ± 3.43			
	5 M^a^	6.82 ± 2.23	7.45 ± 2.12	13.41 ± 2.78			

Lump size	Baseline^b^	6.70 ± 2.54	7.79 ± 3.33	6.82 ± 2.61	144.561/< 0.001	145.131/< 0.001	287.328/< 0.001
	1 M^a^	4.12 ± 1.53	5.15 ± 1.63	6.12 ± 1.93			
	3 M^a^	2.43 ± 0.84	3.41 ± 1.14	4.79 ± 1.67			
	5 M^a^	0.48 ± 0.13	1.31 ± 0.41	2.39 ± 1.24			

*Note:* HAMD = Hamilton Depression Rating Scale, HAMA = Hamilton Anxiety Rating Scale.

Abbreviation: VAS = Visual Analogue Scale.

^a^Intergroup differences are statistically significant.

^b^Intergroup differences are not statistically significant.

**Table 3:** Therapeutic efficacy comparison among three patient groups.

Item	CHQGT combination group (*n* = 160)	Glucocorticoid combination group (*n* = 160)	Control group (*n* = 160)	*χ* ^2^/*F* value	*p* value
Cured	100	99	48	55.824	< 0.001
Improved	47	41	60		
Ineffective	13	20	52		
Effective	91.88%^b^	87.50%^c^	67.50%		

Disease duration (months)	5.54 ± 3.03^ab^	5.85 ± 3.04^c^	8.24 ± 3.21	11.360	< 0.001

^a^Comparison between CHQGT combination group and glucocorticoid combination group, *p* < 0.05.

^b^Comparison between CHQGT combination group and control group, *p* < 0.05.

^c^Comparison between glucocorticoid combination group and control group, *p* < 0.05.

**Table 4:** Comparison of adverse reactions during treatment among three patient groups.

Item (adverse reactions)	CHQGT combination group (*n* = 160)	Glucocorticoid combination group (*n* = 160)	Control group (*n* = 160)	*χ* ^2^ value	*p* value
Skin allergy	0	3	8	29.312	< 0.001
Weight gain	0	6	8		
Diarrhoea	0	6	12		
Local itching	0	0	3		
Skin redness and swelling	4	0	3		
Total incidence	2.50%^a^	9.38%^b^	21.25%		

^a^Pairwise comparison between CHQGT combination group and control group, *p* < 0.05.

^b^Comparison between glucocorticoid combination group and control group, *p* < 0.05.

**Table 5:** Comparison of breast appearance among patients after treatment in three groups.

Item	CHQGT combination group (*n* = 160)	Glucocorticoid combination group (*n* = 160)	Control group (*n* = 160)	*χ* ^2^ value	*p* value
Excellent	58	35	15	45.366	< 0.001
Good	77	68	63		
Fair	22	33	47		
Poor	3	24	35		
Good-to-excellent rate	84.38%^ab^	64.38%^c^	48.75%		

^a^Comparison between CHQGT combination group and glucocorticoid combination group, *p* < 0.05.

^b^Comparison between CHQGT combination group and control group, *p* < 0.05.

^c^Comparison between glucocorticoid combination group and control group, *p* < 0.05.

## Data Availability

The datasets used and analyzed during the current study are available from the corresponding author on reasonable request.

## Nomenclature

ALB: Albumin
BMI: Body mass index
BP: Biological process
CC: Cellular component
CCL2: C-C motif chemokine ligand 2
CEUS: Contrast-enhanced ultrasound
CHQGT: Chai Hu Qing Gan Tang
CRP: C-reactive protein
CXCL8: C-X-C motif chemokine ligand 8
DL: Drug likeliness
HAMA: Hamilton Anxiety Rating Scale
HAMD: Hamilton Depression Rating Scale
IFNG: Interferon gamma
IGM: Idiopathic granulomatous mastitis
IL: Interleukin
MF: Molecular function
MWA: Microwave ablation
NEUT: Neutrophils
NF-κB: Nuclear factor kappa B
OB: Oral bioavailability
PGE2: Prostaglandin E2
PPI: Protein–protein interaction
STAT3: Signal transducer and activator of transcription 3
TCMSP: Traditional Chinese medicine systematic pharmacological analysis platform
TNF: Tumour necrosis factor
VAS: Visual Analog Scale
WBC: White blood cell count
β-EP: Beta-endorphin
